# Grammatical gender in spoken word recognition in school-age Spanish monolingual and Spanish–English bilingual children

**DOI:** 10.3389/fpsyg.2024.1295379

**Published:** 2024-07-24

**Authors:** Alisa Baron, Katrina Connell, Daniel Kleinman, Lisa M. Bedore, Zenzi M. Griffin

**Affiliations:** ^1^Department of Communicative Disorders, University of Rhode Island, Kingston, RI, United States; ^2^Independent Researcher, Florence, SC, United States; ^3^Yale Child Study Center, Yale University, New Haven, CT, United States; ^4^Department of Communication Sciences and Disorders, Temple University, Philadelphia, PA, United States; ^5^Department of Psychology, University of Texas at Austin, Austin, TX, United States

**Keywords:** grammatical gender, bilingualism, eye tracking, visual word paradigm, typically-developing children

## Abstract

This study examined grammatical gender processing in school-aged children with varying levels of cumulative English exposure. Children participated in a visual world paradigm with a four-picture display where they heard a gendered article followed by a target noun and were in the context where all images were the same gender (same gender), where all of the distractor images were the opposite gender than the target noun (different gender), and where all of the distractor images were the opposite gender, but there was a mismatch in the gendered article and target noun pair. We investigated 51 children (aged 5;0–10;0) who were exposed to Spanish since infancy but varied in their amount of cumulative English exposure. In addition to the visual word paradigm, all children completed an article–noun naming task, a grammaticality judgment task, and standardized vocabulary tests. Parents reported on their child’s cumulative English language exposure and current English language use. To investigate the time course of lexical facilitation effects, looks to the target were analyzed with a cluster-based permutation test. The results revealed that all children used gender in a facilitatory way (during the noun region), and comprehension was significantly inhibited when the article–noun pairing was ungrammatical rather than grammatical. Compared to children with less cumulative English exposure, children with more cumulative English exposure looked at the target noun significantly less often overall, and compared to younger children, older children looked at the target noun significantly more often overall. Additionally, children with lower cumulative English exposure looked at target nouns more in the different-gender condition than the same-gender condition for masculine items more than feminine items.

## 1 Introduction

Children and adults process spoken language incrementally, utilizing the partial information at any given moment to predict upcoming words based on lexical or morphosyntactic cues, among others (e.g., Marslen-Wilson, 1987; Bates et al., 1996; Friederici and Jacobsen, 1999; Fernald et al., 2001). Grammatical gender is one example of such a cue that both children and adults efficiently use in spoken word recognition (or visual world paradigm) (Dahan et al., 2000; Lew-Williams and Fernald, 2007). The present study investigates spoken word recognition in school-aged children who speak Spanish and the influence of cumulative English exposure on this ability.

### 1.1 Spanish grammatical gender

Languages such as Spanish, French, and Dutch (and many others) assign grammatical gender to nouns. The gender of a given noun impacts the surface form of other words in the sentence that modify it, such as adjectives and determiners, so that they agree in gender and number. Spanish has two genders: masculine and feminine. Spanish nouns have a reliable pattern of overt gender marking, with masculine nouns ending in –*o* 99.9% of the time and feminine nouns ending in –*a* 96.6% of the time (Teschner and Russell, 1984). Although there are nouns that do not follow this reliable pattern, here, our focus is on nouns with canonical -o/-a endings. Definite and indefinite articles in Spanish are among the most frequently used words, and they agree in gender and number with the noun they modify. Singular masculine nouns are preceded by “*el*” or “*un*,” and singular feminine nouns are preceded by “*la*” or “*una*.” This regularity of the Spanish gender system facilitates early monolingual child acquisition of grammatical gender in Spanish articles (Perez-Pereira, 1991).

### 1.2 Acquisition and use of grammatical gender in monolinguals

For monolingually Spanish-exposed children, grammatical gender emerges in production at approximately 1;6 (year;months) (Hernández-Pina, 1984; Lleó, 1998), and they master gender agreement in production by age 3 (Soler, 1984; Perez-Pereira, 1991; López-Ornat, 1997; Eisenchlas, 2003; Castilla and Pérez-Leroux, 2010; Mariscal and Auza, 2017). Spanish–English bilinguals, Spanish speakers with a developmental language disorder (DLD), and Spanish heritage speakers produce more gender errors than their age-matched monolingual peers, which suggests that their acquisition differs from that of typically developing, monolingually-exposed children (Bruhn De Garavito and White, 2002; Bedore and Leonard, 2005; Morgan et al., 2013; Cuza and Pérez-Tattam, 2015).

In online language comprehension tasks, toddlers (2–3 years old) learning Spanish as their first language use morphosyntactic markers of grammatical gender to rapidly identify visual referents. Monolingual Spanish-speaking children take advantage of gender-marked words in real time to interpret spoken sentences rapidly (Lew-Williams and Fernald, 2007). In the study by Lew-Williams and Fernald (2007), children were presented with two objects while they listened to a speaker name one of the objects using an article + noun combination. Their task was to look at the picture that is named during a pre-recorded sentence (i.e., “Where’s the [ball]? and Do you see it?”). The experiment included two conditions: a different-gender condition and a same-gender condition. For the same-gender trials, the two presented objects had the same grammatical gender, thus requiring the listener to wait for the noun to ascertain which object was being referred to. For the different-gender trials, the two presented objects differed in their grammatical gender, thus allowing the article to be predictive of the upcoming referent noun. Children’s eye movements were tracked as they performed the task, and the results showed that these monolingual Spanish-speaking children oriented faster to the correct referent on different-gender trials where the article was informative, compared to the same-gender trials where the article was uninformative. This study indicated that adjacent, informative, grammatical cues (such as a gendered article) are used to facilitate online speech comprehension.

Similarly, preschool (5;4–6;6) and first grade (6;5–7;7) children in Chile were shown 32 distinct visual displays with four pictures of familiar objects at a time, each with a pre-recorded article–noun phrase matching one of the pictures (i.e., !Mira La manzana [Look! The_ART.F.SG_ apple]) (Coloma et al., 2023). Researchers investigated whether children would use grammatical gender predictively in regard to definiteness (definite/indefinite), gender (masculine/feminine), and number (singular/plural). Children heard different combinations of definite (i.e., *el, la, los,* and *las*) and indefinite (i.e., *un, una, unos,* and *unas*) articles. These children tended to look more at the target object compared to the surrounding competitors even before the target object was mentioned (Coloma et al., 2023). In this longitudinal study, preschoolers showed a small but reliable anticipation effect to the target noun (~150 ms after the onset of the target noun) and, once in first grade, attended to the target noun ~300 ms before it was even mentioned. Within a similar design, bilingual Spanish–Catalan children (4;6–12;2) in Spain also saw four objects with conditions varying by definiteness, number, and gender with a pre-recorded sentence matching one of the objects (i.e., La chica muerde la manzana [The girl bites the apple]). Children were also able to identify the correct target before the target noun was stated based on the preceding gender-marked article (Christou et al., 2020).

### 1.3 Use of grammatical gender in bilingual children

Early bilingual acquisition of grammatical gender has been shown to be modulated by exposure and language dominance in simultaneous (exposure to both languages from birth) and sequential (exposure to a second language after age 3) bilingual children (e.g., Cornips and Hulk, 2008; Unsworth, 2013; Unsworth et al., 2014; Rodina and Westergaard, 2017). Studies on gender processing in bilingual children are quite sparse. Children (aged 8–9) who learn languages simultaneously, where both languages utilize grammatical gender, appear sensitive to grammatical gender cues (Lemmerth and Hopp, 2019). However, it appears that there are cross-linguistic influences at play and a possible influence of age of acquisition (i.e., simultaneous vs. sequential bilinguals). In another study of sequential Mandarin–Italian bilinguals, researchers found that a subset of the Mandarin–Italian bilinguals were slower or did not use grammatical gender predictively at all when compared to Italian-speaking monolingual peers (Bosch et al., 2022). They noted that gender processing was significantly affected by proficiency in the second language, Italian. Finally, in a study of 8- to 12-year-old early bilingual and multilingual children learning Italian in a majority context, there was a clear anticipatory gender effect, but multilingual children were slower than their Italian-speaking monolingual peers (Bosch and Foppolo, 2023).

In the US, many bilingual children of immigrant parents, who are referred to as heritage speakers, learn an ethnolinguistically minority language (Silva-Corvalán, 1994; Kondo-Brown, 2006). Within the US, many children who are heritage speakers of Spanish begin their formal education as Spanish-dominant speakers. However, these children quickly transition to become English-dominant due to their increased exposure to English in school. As time goes on and they progress in school (typically English only), they learn to read only in English, and the amount of input in Spanish decreases and becomes mostly limited to use at home (Montrul et al., 2008; Montrul, 2016). This decreased input and lack of literacy in Spanish can negatively impact these children’s grammatical skills, leading to more grammatical errors (Montrul et al., 2008; Polinsky, 2008). These errors are often in areas that are particularly vulnerable for heritage speakers (i.e., gendered articles, direct object clitics, and subjunctive; Anderson, 1999a,b; Guiberson et al., 2015). Basic morphosyntactic structures are typically acquired by ages 4–5 for monolinguals (e.g., Brown, 1973; Castilla, 2008), but for bilinguals, this is a vulnerable time as children are still acquiring both of their languages and the reduced exposure to Spanish may have consequences on their grammar acquisition. Several researchers have noted that even by 6.5, many bilinguals in the US have still not acquired gendered articles expressively (Morgan et al., 2013), especially those who are more English-dominant bilinguals (Baron et al., 2018). However, there are a few studies that have shown that gendered articles are acquired by Spanish-dominant bilinguals by age 6 (Castilla-Earls et al., 2016) or when they have reached a mean utterance length of 6 (Baron et al., 2018).

Research on US heritage speakers of Spanish has mainly focused on adults (Polinsky, 2008; Montrul, 2016), with few studies focusing on child populations (e.g., Cuza and Pérez-Tattam, 2015; Baron et al., 2018, 2022; Castilla-Earls et al., 2020, 2023). Some literature suggests that heritage Spanish-speaking children do not lag behind their monolingual peers in acquiring gender up to first grade (Snyder et al., 2001; Gathercole, 2002; Kuchenbrandt, 2005). One study showed that older children, in second and third grades, appeared to have an asymmetry where they were more accurate in producing masculine agreement than feminine (Sadek, 1975). Another study showed younger children (6- to 8-year-olds) produced agreement errors, while older children (9- to 11-year-olds) produced no errors in agreement (Montrul and Potowski, 2007). Due to the variability in findings, the developmental path that heritage Spanish-speaking children take in the acquisition of specific grammatical properties continues to be unclear. Montrul et al. (2008) concluded that heritage speakers have competence in grammatical gender and agreement but that the task modality and the type of linguistic knowledge that must be established affect heritage speaker’s performance due to the nature of their language acquisition experience.

To account for the observations noted previously in regard to gender sensitivity, multiple theories make predictions about the ways that listeners will respond to gender cues. One can consider grammatical gender in terms of the Competition Model, in which the utility of a cue’s strength varies as a result of learning and processing (MacWhinney, 1987). Forms are initially transferred on the basis of their ability to apply to new cases. However, if this transfer leads to an error or is unnecessary, the strength of the transfer is weakened. As English does not have grammatical gender, the strength of the cue may be weakened. Four patterns emerge when considering cue strength: (1) transfer of the first language (L1) onto the second language (L2), (2) abandonment of L1 for L2, (3) merger of L1 and L2, and (4) partial attainment of separate L1 and L2 systems. The merger of L1 and L2 or partial attainment of separate L1 and L2 systems is the pattern that is most likely to affect gender processing. Early on in learning, the concept being expressed (gender sensitivity) would be more strongly associated with the form consistent with the L1 contingencies (e.g. *el gato* [the.MASC cat]) than with the form consistent with the L2 contingencies (e.g. *the cat*) (Trenkic, 2009). With more L2 experience, where gender is not marked and there is no full nominal gender system, the strength of the association in the L2 (*the cat*) is likely to increase. Another factor determining the outcome of the competition is the cognitive architecture and mechanisms involved in language processing, as well as the capacity-limited nature of working memory (MacWhinney et al., 1984). The details of the mental representation of “*el gato*” versus “*the cat*” differ across models of the mental lexicon and lexical processing. Under the Competition Model, the two competing referential forms differ in terms of their complexity, with “*el gato*” being structurally more complex than “*the cat*” due to the additional gender component. Other concurrent processes and representations may restrict the resources available for referential processing. The more demand other processes make on the limited resources, the more likely it is that they will encroach on the space needed for gender processing. This, in turn, will cause the processing of the more complex expression (*el gato*) to become increasingly unaffordable or unnecessary and leave the simpler form (*the cat*) the winner. As more cognitive resources become available with increased proficiency, there will be fewer instances in which resources are exceeded to the extent that they will completely preclude the processing of the more complex expression (*el gato*). Thus, proficiency/current language use may lead to predictable patterns of gender sensitivity. If the L1 and L2 merge, within this process, we would expect to see changing levels of gender processing and accuracy.

#### 1.3.1 Gender asymmetry

To further expand on bilinguals’ use of grammatical gender, some researchers have found an asymmetry between the use of masculine- and feminine-gendered articles. Although many researchers have not observed an asymmetry between genders, this phenomenon has been typically explained in regard to the masculine default hypothesis in which the masculine gender is considered the default or unmarked gender in Spanish (Harris, 1991), French (Hulk and Tellier, 1999), Greek (Tsimpli and Hulk, 2013), Italian (Riente, 2003), and more. Feminine gender agreement seems to be more recognizable or salient in a variety of online and offline tasks when compared to the default masculine gender (Domínguez et al., 1999; Alemán Bañón and Rothman, 2016; Beatty-Martínez and Dussias, 2019; Hur et al., 2020). Perhaps, since there is reduced input and output of the heritage language, speakers then overextend the masculine gender marking in gender agreement (Cuza and Pérez-Tattam, 2015). Within eye-tracking tasks, Spanish–English-speaking adults have been shown this gender asymmetry (to use the feminine article to facilitate processing but no facilitative process for the masculine article) (Valdés Kroff et al., 2017). Valdés Kroff et al. (2017) stated that this asymmetry may be due to an extensive overuse of the masculine gender in code-switching (as the asymmetry was noted during code-switched trials), which in turn leads speakers to ignore the gender cue during comprehension. Gender asymmetry has also been recently documented in bilingual Spanish–English-speaking children (Baron et al., 2022), where children used the feminine gender during facilitatory processing but not the masculine gender. Therefore, it seems that there is a distinction in how masculine and feminine gender are represented and processed in Spanish, stemming from distributional asymmetries (Beatty-Martínez and Dussias, 2019). In sum, it appears that the processing of grammatical gender in Spanish does indeed vary.

### 1.4 Grammatical gender mismatch

In addition to examining the processing of grammatical article–noun pairings, testing the processing of ungrammatical article–noun pairings, or gender mismatches, can also be informative in the study of language acquisition. Gender mismatches occur when the gender of an article and the adjacent noun are mismatched (e.g., *el_ART.M.SG_ [the] *pelota_F.SG_* [ball]) and therefore are ungrammatical. Complementary to the study of processing with grammatical pairings, which focuses on the facilitatory effect of articles on their following noun, the study of gender mismatches can reveal sensitivity to ungrammaticality by showing inhibition. Therefore, even though it is effortful to recognize grammatical uninformative nouns, it is even harder to overcome ungrammatical article–noun pairings as it impedes comprehension for a longer time (Dahan et al., 2000; van Heugten and Shi, 2009).

Researchers who have studied the processing of gender mismatches in several gendered languages have all shown delayed noun recognition and significant inhibitory effects in both children and adults in online and offline tasks (e.g., Colé and Segui, 1994; Bates et al., 1996; Jakubowicz and Faussart, 1998; Faussart et al., 1999; Jacobsen, 1999; Dahan et al., 2000; Wicha et al., 2005; Lew-Williams and Fernald, 2007; van Heugten and Shi, 2009). In several studies, 2-year-old children exposed to French showed delayed and inhibited recognition of nouns when the article and noun were mismatched compared to when they matched (e.g., van Heugten and Shi, 2009; van Heugten and Johnson, 2011). Additionally, Guillelmon and Grosjean (2001) conducted a study with English–French adult bilinguals and monolingual French speakers. Nouns were preceded by a correct, an incorrect, or a neutral gender-marked article, and participants were asked to listen to an article–adjective–noun phrase and repeat the noun as quickly as possible. Early English–French bilinguals behaved like monolinguals in their sensitivity to gender (in matched versus mismatched trials). Late bilinguals, on the other hand, did not show a matched versus mismatched effect even when controlling for the speed of response and gender-production skills. Within an acceptability judgment task, Gómez Carrero and Ogneva (2023) found that both Russian-speaking and English-speaking L2 learners of Spanish were sensitive to gender mismatches. They speculated that an “overt morphology on the noun may act as a gender cue and facilitate the detection of gender mismatches.” Thus, if ungrammatical article–noun pairings significantly impede comprehension, this may demonstrate that some article–noun dependencies have been learned and can constrain possible word candidates.

### 1.5 The present study

The present study examines the influence of cumulative language exposure (the number of years a child has been exposed to a language) on the processing of grammatical gender in monolingual Spanish-speaking and bilingual Spanish–English-speaking children by testing grammatical and ungrammatical article–noun pairings. In the visual world paradigm, participants are presented with a visual scene, and eye movements are recorded as they hear instructions to identify or manipulate objects on a screen. Using the visual world paradigm and behavioral measures, the present study addresses the following questions in monolingual Spanish-speaking and bilingual Spanish–English-speaking children aged 5 to 10 years old: Does cumulative language exposure to English reduce the use of gender as a cue to facilitate processing in Spanish in school-aged children? If so, do children show a differential use of gender (masculine vs. feminine)?

## 2 Materials and methods

### 2.1 Participants

Thirty monolingual Spanish-speaking children from Querétaro, Mexico, and 34 bilingual Spanish–English-speaking children from Austin, Texas, were recruited. All parents and children gave informed consent/assent to participate in the study and were compensated for their participation. This study was approved by the Institutional Review Boards at the University of Texas at Austin and the University of Rhode Island. Only participants who met the following criteria were recruited for the study: (1) ages 5–10, (2) exposure to Spanish from birth, (3) no focal brain injury, severe social–emotional problems, genetic syndromes, intellectual disability, autism spectrum disorder, hearing loss, and speech or language disorders as reported by parents, and (4) normal hearing and normal/corrected vision as reported by parents. Additionally, children were included in the study if they completed the eye tracking task (N = 13 excluded due to difficulty tracking their eye movements). Thus, 51 participants (19 F, 24 M, 8 unreported) were included in the final analyses for this study, which included 25 children from Mexico and 26 children from Texas.

### 2.2 Behavioral measures

#### 2.2.1 Bilingual input–output survey (BIOS)

To examine a child’s communication abilities in the language(s) spoken, parents completed the *Bilingual Input–Output Survey* (BIOS) in person, a questionnaire subtest within the *Bilingual Spanish English Assessment* (BESA; Peña et al., 2018). Parents detailed the history of exposure to each language a child speaks at home and school environments since birth to calculate the child’s age of first exposure to English and language(s) spoken at each year of the participant’s life. Parents also reported the current language input and output at home and school on an hourly basis during the week and on weekends. Cumulative English exposure is defined as the number of years a child hears and speaks English from the first year they begin to be exposed to English.

#### 2.2.2 Expressive one-word picture vocabulary test (EOWPVT)

The Expressive One-Word Picture Vocabulary Test-Fourth Edition (EOWPVT-4; Martin and Brownell, 2011) and the Expressive One-Word Picture Vocabulary Test-4: Spanish Bilingual Edition (EOWPVT-4 SBE; Martin, 2013) are norm-referenced tests of single-word expressive vocabulary that were used to provide a gross measure of cumulative vocabulary knowledge in each language that a child speaks. After signing consent, each test was administered one time to all participants. For the current study, the EOWPVT-4 and EOWPVT-4 SBE were administered as English-only and Spanish-only versions, respectively. If the participant responded in the non-target language, the examiner redirected and prompted the participant to respond in the target language. Basal and ceiling were achieved based on the directions outlined in the test manuals for each test. The ceiling rule for the EOWPVT-4 is six consecutive incorrect responses which were especially relevant for monolingual Spanish speakers as they had very little or no exposure to English. Raw scores were calculated for each language.

#### 2.2.3 Article–noun pair naming task

Upon conclusion of the EOWPVT, participants completed a familiarization task, which included 234 images used in the eye-tracking experiment (explained in more detail in Section 2.3). Participants were instructed to name each picture in Spanish with its corresponding definite article (el_ART.M.SG_ or la_ART.F.SG_). This task was included to examine whether participants were able to assign the correct/target name and gender to each image. Participants were only provided with the correct response (article+noun) if they did not know the item. If they provided a non-target name (i.e., el_ART.M.SG_ coche [the car] for el_ART.M.SG_ carro [the car]), they were prompted to label the item again. Participants were not asked to repeat the target name after the model was given, but many did so spontaneously. Each response was recorded, and participants’ production accuracy on gender-marked articles was calculated where a score of 1 was given to correctly named images (both article and noun) and a score of 0 for an incorrectly named image (article or noun) or if the participant was unable to name the image.

### 2.3 Eye-tracking task

#### 2.3.1 Materials

Thirty-six familiar nouns in Spanish (18 masculine and 18 feminine) were included as targets in the experimental stimuli. Twelve filler items were included for a total of 48 stimuli. Two practice items were presented at the beginning of the task to allow participants to become accustomed to the nature of the experiment. The target location was counterbalanced such that target stimuli appeared in each quadrant on the screen the same number of times.

Selection of noun targets and phonological competitors was restricted to words with the same initial consonant–vowel. The phonological competitor in each item had a similar syllable length to the target noun (+/− 1 syllable). The other two distractors in each stimulus also began with consonants (except for /l/ as in connected speech, the /l/ in the article “el” tends to be elongated to include the initial /l/ of the target noun becoming one word rather than two distinct words). Three presentation lists were created so that across participants, each target stimulus occurred in each condition (different, same, and ungrammatical) across lists (according to a Latin square design). The two distractors in each stimulus used for the different gender and ungrammatical gender conditions stayed together for a different target stimulus in the same-gender condition across presentation lists. Additionally, items from different categories (e.g., animals, foods, furniture, transportation, musical instruments, and clothing) were not presented together with the exception of cuchillo/cuchara [knife/spoon], pavo/pato [turkey/duck], tiburón/tigre [shark/tiger], zanahoria/salsa [carrot/salsa], and canguro/caballo [kangaroo/horse], to avoid semantic competition effects (e.g., Huettig and Altmann, 2005). However, these five items were included as they were phonological competitors with the same initial consonant–vowel and were the most closely matched selections based on noun frequency. In addition, animals are high-frequency words that most 5-year-old children are familiar with, and thus, some of the distractors were animals, even if the target or phonological competitor was an animal.

Based on the Spanish Lexical Database (Espal, Duchon et al., 2013), the phonological competitors had an average lexical frequency that was higher (*M* = 1.13, *SD* = 0.70, range = 0.08–3.33) than the frequency of the target nouns (*M* = 1.01, *SD* = 0.54, range = 0.04–2.34). The phonological competitor lexical frequency between presentation lists was not significantly different (*p*s > 0.12 in pairwise *t*-tests). The distractors’ lexical frequency was similar across the same (*p*s > 0.12), different (*p*s > 0.26), and ungrammatical (*p*s > 0.26) gender conditions between presentation lists.

One hundred and eleven colored pictures were selected from Snodgrass and Vanderwart (1980), and an additional 123 highly imageable and concrete items were also selected. Across all three presentation lists, there were a total of 234 pictures. A subset of the items had imageability, concreteness, and familiarity ratings using EsPal (81% of the feminine items, 70% of the masculine items). Imageability, concreteness, and familiarity all use a scale from 1 to 7, with 7 indicating fully imageable, concrete, or familiar. The feminine items had average ratings of 6.16, 5.88, and 6.07, and the masculine items had average ratings of 6.16, 5.99, and 5.89 on imageability, concreteness, and familiarity, respectively. Ratings across feminine and masculine items were not significantly different. Pictures were normed for naming agreement by the first author with 10 adult and 6 child heritage Spanish speakers. Pictures with above 80% agreement were used as targets, while pictures below 80% were used in filler items. The pictures were edited to fit within 462 × 334 pixels.

**Figure 1:**
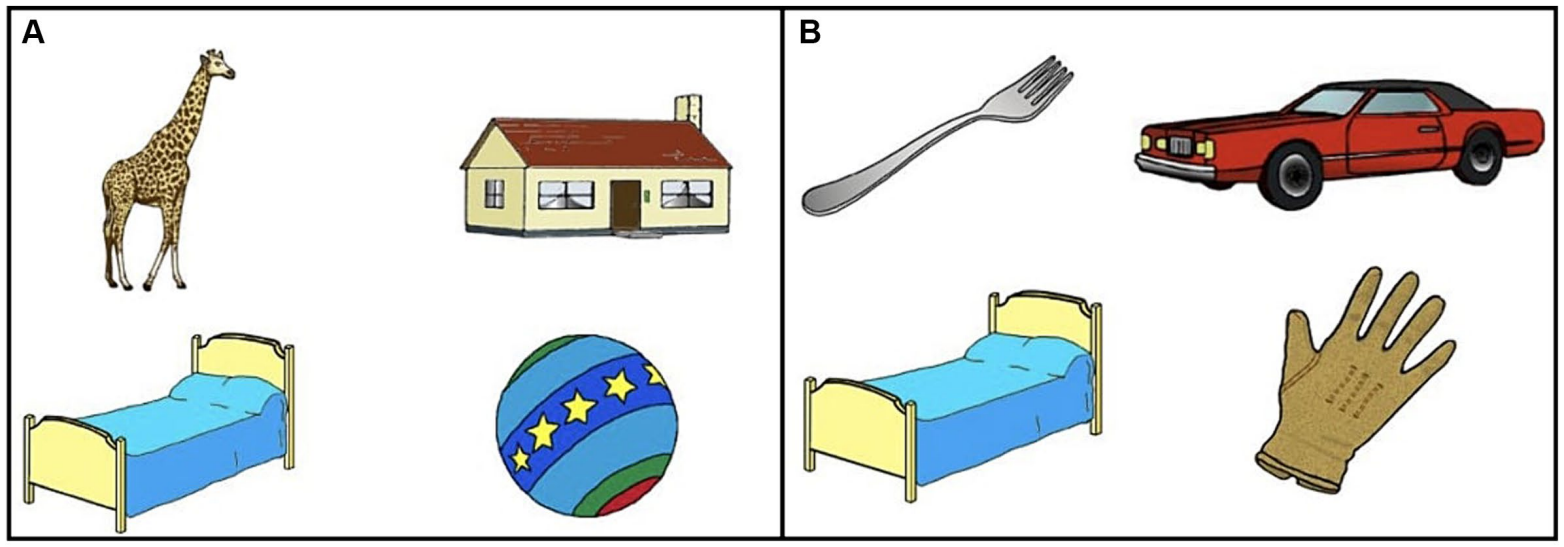
Examples of **(A)** same gender (e.g., la cama [fem.bed] as the target), **(B)** different gender (e.g., la cama [fem.bed] as the target), and ungrammatical gender (e.g., *el cama [masc.bed] as the target) displays including la cama [fem.bed] as the target noun.

Participants heard the sentence “*enséñame* + *el/la* + *target noun*” [show me + the + target noun]. A bilingual male speaker of Mexican Spanish recorded each sentence. For sentences that were ungrammatical, the article was spliced from a grammatical sentence with the same initial consonant of a different target noun and was inserted into the ungrammatical sentence so that the sentence sounded natural. The articles “*el*” and “*la*” were unstressed within the sentence. “Enséñame [show me]” was used as the instruction because in Spanish, if one says “mira a [look at] + el”, the “a + el” is combined to form “al” where “a + la” stays unchanged. Therefore, “enséñame”, which has been used in previous gender processing studies, was selected for the instructions (e.g., Lew-Williams and Fernald, 2007, 2010; Baron et al., 2022). For example, the participants heard ‘enséñame la cama’ [show me the_ART.F.SG_ bed_F.SG_], where “*la cama*” was the target (for all three conditions described below). For the same-gender condition, “*la casa*” [the_ART.F.SG_ house_F.SG_] was the phonological competitor, and *la pelota* [the_ART.F.SG_ ball_F.SG_] and *la jirafa* [the_ART.F.SG_ giraffe_F.SG_] were the distractors (Figure 1A). For the different gender condition, *el carro* [the_ART.M.SG_ car_M.SG_] was the phonological competitor, and *el guante* [the_ART.M.SG_ glove_M.SG_] and *el tenedor* [the_ART.M.SG_ fork_M.SG_] were the distractors (Figure 1B). For the ungrammatical condition, participants heard “enséñame *el cama” [show me the_ART.M.SG_ bed_F.SG_], where *la cama* was still the target, *el carro* was the phonological competitor, and *el guante* and *el tenedor* were the distractors (Figure 1B). Although the competitor and distractor pictures were the same for each item in the different-gender and ungrammatical conditions, the auditory stimulus was different. The different-gender condition had a target that was grammatical (*la cama*), while the ungrammatical condition had a target that was ungrammatical (*el cama).

#### 2.3.2 Design and procedure

Three groups of experimental stimuli were prepared: one group with informative (different-gender) articles, one group with uninformative (same-gender) articles, and one group with incorrect (ungrammatical) articles. A target appeared only once in each presentation list, preceded by the correct (or incorrect) gender marking surrounded by distractors with the same or different gendered articles. There were three presentation lists, and each target stimulus occurred in each condition across the lists. For example, “el conejo” [the rabbit] occurred in the same-gender condition in List 1, the different-gender condition in List 2, and the ungrammatical condition in List 3. Each participant completed one presentation list.

The experiment was built using EyeLink Experiment Builder software (v2.1.1). In Texas, eye movements were recorded on an EyeLink 1000, while in Mexico, eye movements were recorded on an EyeLink Portable Duo due to its portability. SR Research produces both eye trackers. All procedures were the same across locations. The sample rate was 500 Hz. Viewing was binocular, but eye movements were recorded monocularly. Participants were tested individually in a quiet laboratory space. Stimuli were presented on a 27-in monitor, with participants seated approximately 67 cm from the monitor with chins on a chin rest. If a child could not tolerate the chin rest, a remote tracking setting was used (only available for EyeLink Portable Duo). At the beginning of the task, participants were instructed to use a mouse to click on the object on the screen that was referred to in the sentence. To begin each trial, participants looked at a validation point in the center of the computer screen before the four images were displayed. Participants could not see the images if they did not fixate on the point in the center of the screen. Once they fixated on the point, four pictures appeared on the screen, and 500 ms later, the sentence was presented auditorily. Participants needed to click on a picture to end the trial. If a participant failed to fixate on the validation point, recalibration of the eye tracker was performed before the next trial began. In each trial, fixations were recorded from the onset of the images on the screen until the participant clicked on an image. Latencies were recorded for mouse-click responses.

### 2.4 Grammaticality judgment task

After the eye-tracking task, 36 targets and 12 fillers from the eye-tracking task were presented in 5–10 word sentences. Two practice sentences were presented at the beginning of the task (e.g., *Monté el camello en el desierto* [I rode the camel in the desert]). Half of the sentences were presented as grammatical sentences and half as ungrammatical sentences. Participants were asked to press one of two buttons indicating if the sentence they heard was grammatical or ungrammatical. The grammaticality across the eye-tracking task and grammaticality judgment task were the same to directly compare participants’ ability to identify the grammaticality of the targets offline within sentences. If a target noun was presented in the eye-tracking task as grammatical, participants heard the noun in a simple grammatical sentence within the grammaticality judgment task. There were three grammaticality judgment lists to mirror that of the eye-tracking task, as the grammaticality of the sentence presented depended on the grammaticality of the target in the eye-tracking task. Participants were asked to listen to the sentences carefully and focus on the grammaticality of the sentence. The participant’s button press was recorded for accuracy and reaction time.

### 2.5 Eye-tracking analyses

The eye-tracking data were exported using SR Research Data Viewer software (SR Research). An interest period was set from the beginning of the article until the participant clicked on an image. A Time Course (Binning) report was used to export the data. This report binned time into 10 ms bins, calculated the proportion of fixations to each image within those bins, and excluded samples that fell outside of four predefined interest areas around the images, as well as samples during blinks or saccades. Only trials where the target was correctly selected were exported for analysis (98.5% of trials for included participants).

Analyses were conducted in R (v3.5.3; R Core Team, 2021). After visual inspection of the eye tracking data, all trials with the target item *patineta* were excluded due to outlier patterns of fixations, leaving 1,759 trials. Of these, trials were excluded if the target was not correctly named in the naming task with the article and noun combination (29.2%), as the expected behavior (increased looks to the target over time) would not occur if participants did not know the target name. (Subsequent exclusion percentages in this paragraph are calculated after these exclusions.) Further data cleaning excluded trials in which participants took longer than 10 s to click on a picture (0.8%). Subsequently, trials were excluded if the participant’s reaction time to click was over 2.5 *SD* of the mean click time (measured across participants on a log scale; 1.7%) or if fixation data were not present through the end of the analysis window (1,200 ms; 2.1%). The remaining 1,179 trials included in analyses were balanced between conditions: Across participants, there were between 189 and 210 trials in each of the six combinations of target gender and article gender conditions; individual participants had 3–35 trials across conditions (*M* = 23). (Note that analyses weighted each trial equally rather than weighting each participant equally, so participants with fewer trials were given less weight in analyses.)

Fixations were time-locked to the onset of the article preceding the target noun plus a 200 ms baseline (for the time it takes to plan and launch a saccade; Hallett, 1986). To increase power for the statistical analyses (a cluster permutation test, described below), the 10-ms time bins from 0 to 1,200 ms were collapsed into non-overlapping 50 ms bins; the dependent variable—indicating whether the target picture was fixated—was set to 1 if the target picture was the most-fixated interest area in at least one of the five 10-ms bins, and 0 otherwise. (This rebinning had a minimal effect on the data: Target fixations comprised 30.9% of the 10-ms bins and 34.7% of the 50-ms bins.) Finally, 50 ms time bins in which none of the interest areas were fixated were discarded (19.2% overall; between 14.6 and 22.6% in each condition).

To investigate the time course of lexical facilitation effects, looks to the target picture were analyzed with a cluster-based permutation test. These non-parametric tests were developed for the analysis of MEG/EEG data (Maris and Oostenveld, 2007; Pernet et al., 2011) but have also been applied to other time series data, including fixations in the visual world paradigm (e.g., Barr et al., 2014; see also Ito and Knoeferle, 2023) and control for multiple comparisons across time bins via permutation testing. To perform these tests in R, we used the function *clusterperm.glmer* in the *permutes* package (v2.8; Voeten, 2023). The input to this function is a generalized linear mixed-effects model, with maximal random effects, that is fit to trial- and time bin-level data (one observation for every combination of 50 ms time bin, trial, and participant). Initially, random effects are ordered by their contribution to the model, with likelihood-ratio tests used to determine whether additional random effects account for enough variance to be added to the model. Once the maximally parsimonious model is identified, the cluster test is performed using this model to test the significance of each fixed effect. The output of the test, for each factor tested, is a number of temporal “clusters” (time windows) during which the effect of that factor on fixation rates was maximal, as well as a single *p*-value for that factor: If that *p*-value is below the significance threshold (here, 0.05), then that factor is statistically significant (though the significance of the individual clusters/time bins is never directly tested; see Maris and Oostenveld, 2007 and Sassenhagen and Draschkow, 2019 for details). Several effects yielded clusters that were temporally non-contiguous but separated by only one time bin (50 ms); for reporting purposes, those clusters were combined. For transparency, the cluster mass statistic is reported for each statistically significant effect; where an effect was associated with multiple clusters, this statistic is reported for the largest cluster.

The model given as input for this analysis included a binary dependent variable (as described above, with “successes” indicating looks to the target) and four factors and their interactions. Condition (same-gender vs. different-gender vs. ungrammatical article) was Helmert-coded, such that one Condition predictor represented increased looks to the target in the different-gender condition relative to the same-gender condition, and the other Condition predictor represented increased looks to the target in the grammatical conditions (same-gender and different-gender) relative to the ungrammatical condition. Target Gender was treatment-coded (masculine = −0.5, feminine = +0.5). Cumulative English Exposure, which was originally measured on a scale of 0–10 years, was centered at the sample mean of 3.58 years and linearly scaled. Finally, age was entered as a continuous variable, centered at the sample mean of 7.65 years (range = 5.08–9.92 years). Given these contrast weights, the model intercept represents looks to the target, averaging across the levels of Condition and Gender, for participants who had 3.58 years of cumulative English exposure and were 7.65 years old. The model included these four factors and their interactions as fixed effects. It also initially included random intercepts for participants and items, as well as random slopes for all within-factor variables (for Participants: Condition, Target Gender, and their interaction; for Items: Cumulative English Exposure, Age, Condition, and their interactions). Target fixation rates for children who were 7.65 years old and had 3.58 years of cumulative English exposure, averaging across all three conditions and across both target genders, served as the baseline to which all comparisons were made.

## 3 Results

Table 1 shows participant means and standard deviations for age, cumulative English exposure, age of first exposure to English and Spanish, and mother education based on the Hollingshead (1975) index (a proxy for socioeconomic status) at the time of testing by geographic location. For children in Mexico, age and cumulative English exposure are not correlated (r = 0.07, *p* = 0.75), while for children in Texas, age and cumulative English exposure are moderately correlated (r = 0.61, *p* = 0.001).

**Table 1:** Participant demographics.

	Mexico (*N* = 25)		Texas (*N* = 26)	
	Mean	*SD*	Mean	*SD*
Age (years)	7.50	1.46	7.76	1.77
Cumulative English exposure***	1.39	1.77	5.69	2.30
Age of first exposure to Spanish	0.00	0.00	0.00	0.00
English Input/Output (%)***	1.86	4.85	39.20	10.54
Mother Education^a^^**^	2.96	1.21	4.38	1.88

Group differences using an independent-sample t-test between the children from Mexico and Texas are denoted with *p < 0.05, **p < 0.01, and ***p < 0.001.

a Measured using the following scale: 0 = not applicable or unknown, 1 = less than 7th grade, 2 = junior high school, 3 = partial high school, 4 = high school graduate, 5 = partial college, 6 = standard college or university graduation, 7 = graduate/professional training.

Language measure (EOWPVT, grammaticality judgment, and article–noun pair naming accuracy) mean values are presented in Table 2. Grammaticality judgment accuracy is provided for grammatical and ungrammatical sentences. Grammaticality judgment sensitivity is provided as a *d’* score, and *d’* is used to compare the magnitude of discrimination ability. Here, we use *d’* to compare the magnitude of discrimination between the grammatical trials that were correctly judged to be grammatically correct versus the ungrammatical trials that were correctly judged to be not grammatically correct. The larger the absolute value of *d’*, the stronger the sensitivity. In both groups of children, there appears to be a low sensitivity to grammaticality within a grammaticality judgment task. Additionally, within the article–noun naming task, our sample of children from Mexico produced articles with 86.39% accuracy (*SD* = 5.54), and children from Texas produced articles with 59.66% accuracy (*SD* = 24.90).

**Table 2:** Language measures presented in means and standard deviations.

Language measure	Mexico			Texas		
	Mean	*SD*	Range	Mean	*SD*	Range
EOWPVT English***	55.00	0.00	55–55	93.62	21.62	55–138
EOWPVT Spanish***	123.89	13.63	97–145	93.81	19.41	55–126
Grammaticality Judgment Grammatical Sentences^a^	82.82%	14.98	45.83–100	80.13%	17.49	33.33–100
Grammaticality Judgment Ungrammatical Sentences^b^	51.82%	30.32	0–100	44.55%	29.90	0–100
Grammaticality Judgment Sensitivity (*d’*)	−8.74E-08	1.57	−2.80 to 2.41	−2.18E-07	1.57	−2.77 to 2.75
Article–Noun Naming Task Accuracy (%)***	86.39%	5.54	73.93–94.02	59.66%	24.90	16.67–91.45

Group differences using an independent-sample t-test between the children from Mexico and Texas are denoted with *p < 0.05, **p < 0.01, and ***p < 0.001.

a These data reflect grammatical sentences that were correctly judged to be grammatical.

b These data reflect ungrammatical sentences that were correctly judged to be ungrammatical.

**Figure 2:**
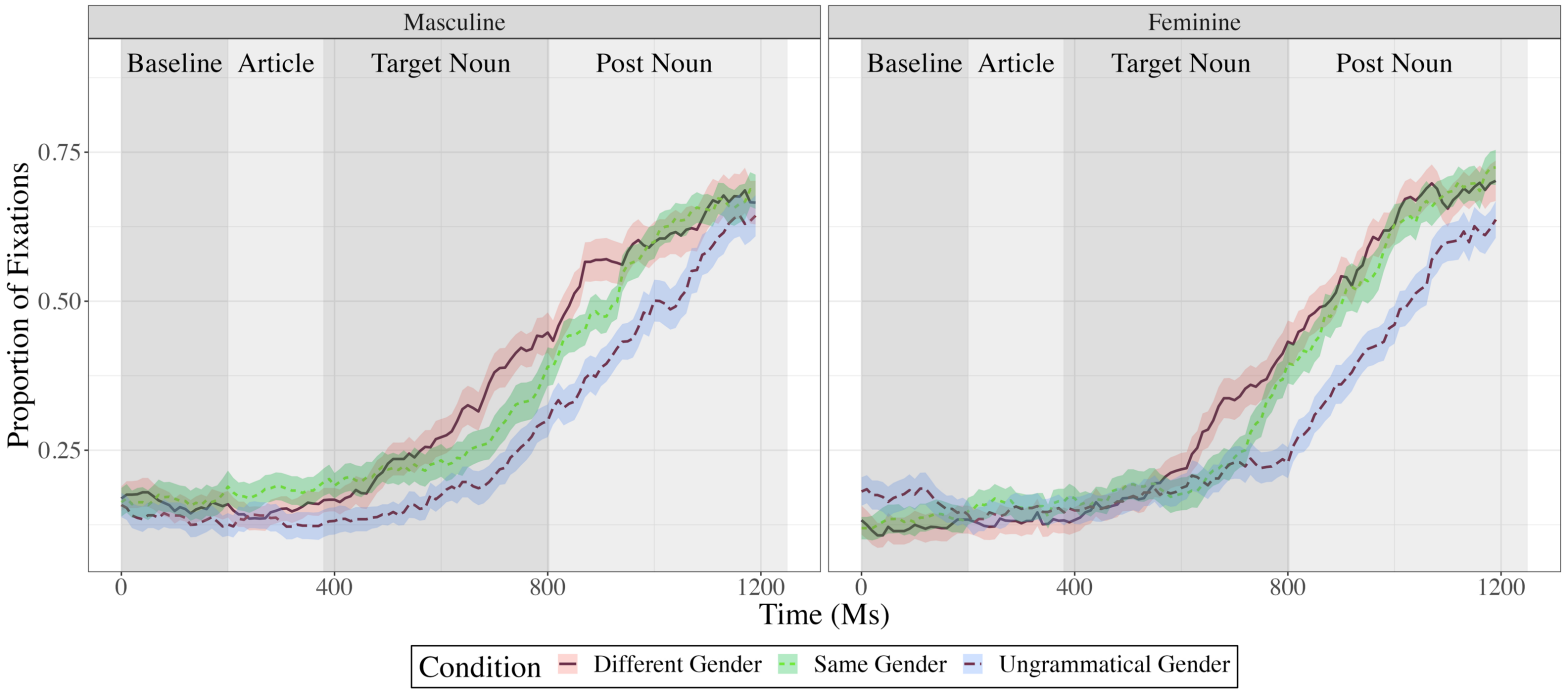
Proportion fixations to target in the different-gender (solid red), same-gender (dotted green), and ungrammatical gender (dashed blue) conditions, as a function of time in milliseconds, separately for targets with masculine gender (left panel) and feminine gender (right panel). Error ribbons represent ±1 standard error of the mean.

**Figure 3:**
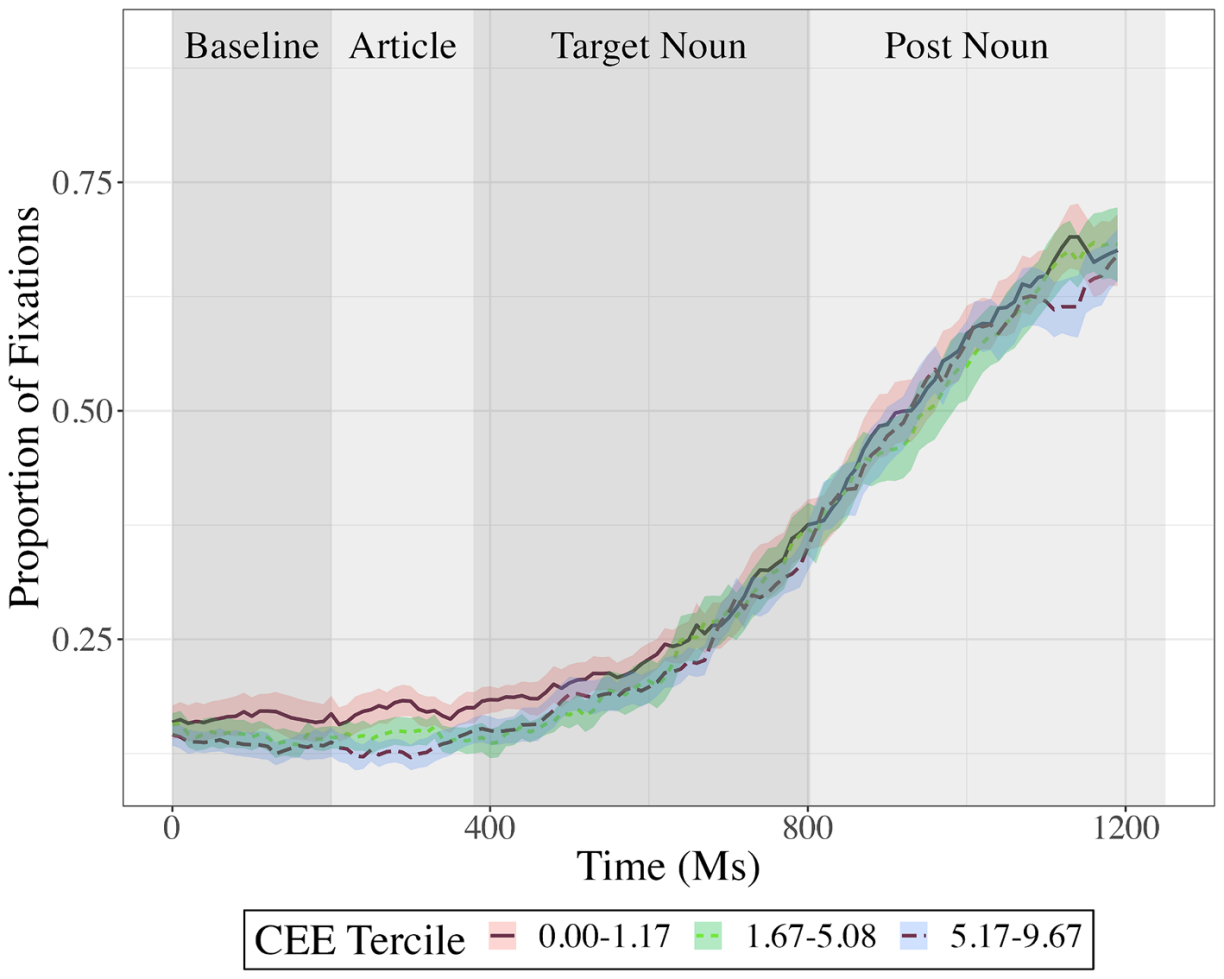
Proportion fixations to target, averaged across conditions and as a function of time in milliseconds, separately for participants based on their cumulative English exposure (CEE) residualized on age: low residual cumulative English exposure (CEE; solid red; n = 17), medium residual CEE (dotted green; n = 17), or high residual CEE (dashed blue; n = 17). Analyses treated CEE as an unresidualized continuous variable; however, participants were grouped into three bins for visualization and residualized to highlight the variance uniquely explained by CEE. Error ribbons represent ±1 standard error of the mean.

**Figure 4:**
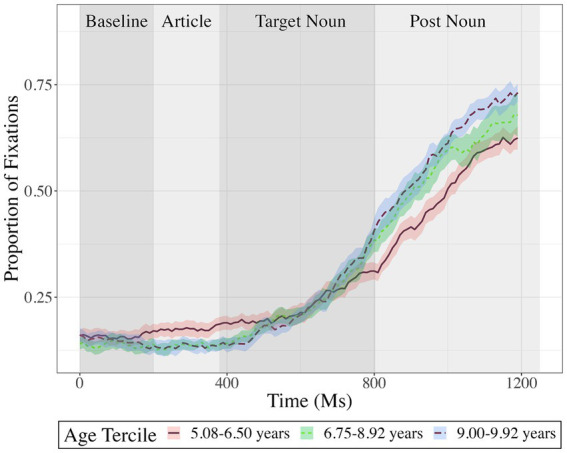
Proportion fixations to target, averaged across conditions and as a function of time in milliseconds, separately for participants based on their age residualized on cumulative English exposure: younger residual age (solid red; *n* = 17), medial residual age (dotted green; *n* = 17), or older residual age(dashed blue; *n* = 17). Analyses treated age as an unresidualized continuous variable; however, participants are grouped into three bins for visualization and residualized to highlight the variance uniquely explained by age. Error ribbons represent ±1 standard error of the mean.

Figure 2 shows fixations to the target object in each experimental condition. Figures 3, 4 show fixations to the target object – averaged across conditions – separately for participants with low, medium, and high residual cumulative English exposure (after regressing out the relationship with age; Figure 3) and residual age (after regressing out the relationship with cumulative English exposure; Figure 4). Both variables were treated as (unresidualized) continuous variables in analyses but were binned and residualized for ease of visualization to reflect the fact that the analysis evaluated the significance of each one while holding the other constant at the sample mean.

Both effects of the condition were significant: Participants looked at the target significantly more often in the different-gender condition than in the same-gender condition, cluster mass = 67, *p* = 0.001, an effect that was maximal from 600 to 1,000 ms. Participants also looked at the target significantly more often in the grammatical conditions than in the ungrammatical condition, cluster mass = 517, *p* = 0.001, an effect that was maximal from 600 to 1,200 ms.

The effect of Target Gender was not statistically significant, *p* > 0.05, indicating no evidence that looks to the target varied as a function of Target Gender. The extent to which participants looked at the target more in the different-gender condition than the same-gender condition was greater for items with masculine gender than items with feminine gender (a two-way interaction), maximal cluster mass = 68, *p* = 0.001, an effect that was present in three non-contiguous temporal clusters from 450 to 550, 800 to 900, and 1,100 to 1,200 ms. Target Gender did not interact with the other effect of condition (grammatical vs. ungrammatical), *p* > 0.05.

Cumulative English exposure and age each showed a significant effect but did not interact with any other factors. Relative to participants with less cumulative English exposure, participants with more cumulative English exposure looked at the target significantly less often overall, cluster mass = 219, *p* = 0.001, an effect that was maximal from 950 to 1,200 ms (see Figure 3). The effect of age was reflected in two clusters with separated time windows and opposing signs (see Figure 4). The first cluster indicated that relative to younger participants, older participants looked at the target significantly less often, with cluster mass = 34, *p* = 0.001, an effect that was maximal at 300 to 550 ms. The second cluster indicated that relative to younger participants, older participants looked at the target significantly more often, with cluster mass = 310 ms, *p* = 0.001, an effect that was maximal from 800 to 1,200 ms.

No other factors were statistically significant (all *p*s > 0.05).

## 4 Discussion

This study investigated the grammatical gender processing of school-aged children growing up in two different language environments: in Mexico, where children were living in a community where Spanish is the dominant language, and in Texas, where children were living in a community where English is the dominant language. Previous researchers have focused on grammatical gender sensitivity to toddlers and adults; however, the literature around child heritage speakers in the US has continued to be limited. In this study, we focused on the influence of cumulative English language exposure in monolingual and heritage speakers of Spanish using grammatical and ungrammatical article–noun pairings. We addressed the research questions using a visual world paradigm in which the gendered article was informative (different gender), uninformative (same gender), or ungrammatical.

First, we examined whether cumulative language exposure to English reduced the use of the grammatical gender cue to facilitate language processing in Spanish. The results showed that all children showed lexical facilitation of informative gender marking on articles to actively anticipate an upcoming word as they looked at the target significantly more often in the different-gender condition than in the same-gender condition toward the end of the noun and during the post-noun region. Lexical processing of word recognition is affected by cumulative English exposure as children with more cumulative English exposure looked at the target noun significantly less often during the article and noun regions than children with less cumulative English exposure. Thus, their accuracy and speed to orient to the target noun were lower than those children with less cumulative exposure. Our findings show that cumulative English exposure does impact the speed and accuracy of online processing.

Additionally, relative to younger children, older children with low cumulative English exposure looked at the target noun significantly more overall during the post-noun region. Thus, older children are more accurate and look to the target nouns faster. Although monolingual children acquire grammatical gender earlier and more accurately than heritage Spanish speakers, all children in this study showed stronger lexical processing when they were older. In previous studies, children showed adult-like processing of grammatical gender but were slower than the adults, potentially due to children’s slower speech processing speed and cognitive resource limitations (Lew-Williams and Fernald, 2007; Snedeker and Huang, 2015; Brouwer et al., 2017). Even though in our study we did not compare children to adults, we do see a similar difference between younger and older children.

In regard to overall grammaticality, all children, regardless of cumulative English exposure, looked at the target more often in the grammatical conditions than the ungrammatical conditions. Children were significantly inhibited by ungrammaticality and showed sensitivity to grammaticality within online processing but struggled during offline processing. Within the grammaticality judgment task, children did not show sensitivity to grammatical gender. This may be due to the fact that children tend to have difficulty with grammaticality judgment tasks as it significantly taxes their working memory capacity (McDonald, 2008). Children generally do not reflect on morphosyntactic structures until middle childhood (Pratt et al., 1984). Until they are older and develop an increased sensitivity to the morphosyntactic structure of sentences, children are more likely to base their judgments on semantic content and pragmatic considerations (or the plausibility of events) than on the grammaticality of the sentence.

Our findings show that children are able to use gender facilitatively but are still acquiring grammatical gender productively. Within the article–noun pair naming task, monolingual Spanish speakers were, on average, 86.39% accurate, while heritage Spanish speakers were 59.66% accurate (with a very large range of 16.67–91.45%). The path to productivity may not be a linear one, as both linguistic input and production relate to and support comprehension through early language development (Chang et al., 2006; MacDonald, 2013).

Furthermore, we investigated whether language processing differed by grammatical gender to better understand if there was a gender bias. Children with lower cumulative English exposure looked at target nouns more in the different-gender condition than the same-gender condition for masculine items more than feminine items. Thus, children with lower cumulative English exposure were facilitated more by the masculine article “*el*” than the feminine article “*la*”. Other researchers have found either no asymmetry or demonstrated a gender asymmetry where they have typically found that feminine is more salient and is used to facilitate processing (Valdés Kroff et al., 2017; Baron et al., 2022). Thus, it is interesting to note that in our study, there is a gender asymmetry; however, the more salient gender appears to be masculine, and thus, children appear to be overusing masculine. Recently, Colantoni and Leroux (2024) noted that although many heritage speakers of Spanish performed at ceiling when grammatical gender was tested, a quarter of the children (aged 4;10–12;7, *M* = 8;08) still displayed a lower accuracy. They attributed this to potentially different gender grammar as some children overuse one gender, meaning they only used masculine or feminine. Within our study, we also anecdotally noticed that some children overused one gender and thus only used masculine or feminine during the article–noun pair naming task. Thus, for a subset of the children, there appears to be a “strong indication of divergence” when considering grammatical gender (Colantoni and Leroux, 2024). Other researchers have posited that heritage speakers may fail to assign any gender. The absence of gender assignment may surface as masculine morphology in Spanish, and thus, heritage speakers may appear to overuse masculine gender. This failure to assign gender may lead heritage speakers to a decreased tolerance for morphophonological irregularity (Fuchs et al., 2021).

### 4.1 Limitations

As noted previously, target nouns that were not named correctly during the article–noun pair naming task were excluded from the cluster-based permutation test. Given that the trial counts were already very low for several participants, the accurate naming of distractors was not considered as an additional exclusion criterion and is thus a limitation of this study.

### 4.2 Conclusion and future directions

In summary, school-aged children showed lexical facilitation of grammatical gender in online processing in that children looked at the target significantly more often in the different-gender condition than in the same-gender condition. Additionally, all children looked at the target significantly more often in the grammatical conditions than in the ungrammatical condition. Children with less cumulative English exposure looked at the target noun significantly more often than children with more cumulative English exposure. In regard to age, older children looked at the target noun significantly more often than younger children. Furthermore, a gender asymmetry was noted where children with less cumulative English exposure looked at the masculine target items more than feminine target items. Moreover, to the authors’ knowledge, this is only the second study to use the visual world paradigm with school-aged children across the Spanish language spectrum to investigate grammatical gender. There continues to be a need to better understand Spanish language acquisition in heritage speakers of Spanish as there is a growing number of Hispanic children learning Spanish as a heritage language. Future work should continue to investigate heritage speakers across the lifespan (especially in children and adolescents) to examine the anticipation and/or facilitation of the gender cue in Spanish.

## Data availability statement

The raw data supporting the conclusions of this article will be made available by the authors, without undue reservation.

## Ethics statement

The study involving humans were approved by the University of Texas at Austin and the University of Rhode Island. The study was conducted in accordance with the local legislation and institutional requirements. Written informed consent for participation in this study was provided by the participants’ legal guardians/next of kin.

## Author contributions

AB: Conceptualization, Data curation, Formal analysis, Funding acquisition, Investigation, Methodology, Writing – original draft, Writing – review & editing. KC: Formal analysis, Visualization, Writing – original draft, Writing – review & editing. DK: Writing – review & editing, Formal analysis. LB: Methodology, Writing – review & editing. ZG: Methodology, Writing – review & editing.
